# Plasma levels of CGRP and expression of specific microRNAs in blood cells of episodic and chronic migraine subjects: towards the identification of a panel of peripheral biomarkers of migraine?

**DOI:** 10.1186/s10194-020-01189-0

**Published:** 2020-10-16

**Authors:** Rosaria Greco, Roberto De Icco, Chiara Demartini, Anna Maria Zanaboni, Elena Tumelero, Grazia Sances, Marta Allena, Cristina Tassorelli

**Affiliations:** 1 Headache Science & Neurorehabilitation, IRCCS Mondino Foundation, Pavia, Italy; 2 Department of Brain and Behavioral Sciences, University of Pavia, Pavia, Italy

**Keywords:** Migraine, CGRP, MicroRNA, Biomarkers, Pain, Medication overuse, Detoxification, Headache

## Abstract

**Background:**

Migraine can manifest with an episodic or a chronic pattern in a continuum of disease severity. Multiple factors are associated with the progression of the pattern from episodic to chronic. One of the most consistently reported factors is the overuse of medications (MO) for the acute treatment of migraine attacks. The mechanisms through which MO facilitates the transformation of episodic migraine (EM) into chronic migraine (CM) are elusive. In order to provide insights into these mechanisms, the present study aims to identify possible peripheral biomarkers associated with the two forms of migraine, and with the presence of MO.

**Methods:**

We evaluated the plasma levels of calcitonin gene-related peptide (CGRP) and the expression of miR-34a-5p and miR-382-5p in peripheral blood mononuclear cells of subjects with EM (*n* = 27) or CM-MO (*n* = 28). Subjects in the CM-MO group were also tested 2 months after an in-hospital detoxification protocol.

**Results:**

CGRP, miR-382-5p, and miR-34a-5p levels were significantly higher in CM-MO subjects when compared to EM patients (*p* = 0.003 for all comparisons). After correcting for age, sex, and disease duration, miRNAs expression was still significantly associated with migraine phenotype (EM vs. CM-MO: *p* = 0.014 for miR-382-5p, *p* = 0.038 for miR-34a-5p), while CGRP levels were not (*p* = 0.115). CGRP plasma levels significantly and positively correlated with miR-382-5p (Spearman’s rho: 0.491, *p* = 0.001) and miR-34a-5p (Spearman’s rho: 0.303, *p* =0.025) in the overall population. In the CM-MO group, detoxification significantly decreased CGRP levels and miRNAs expression (*p* = 0.001). When comparing responders and non-responders to the detoxification, the former group (*n* = 23) showed significantly higher levels of CGRP at baseline, and significantly lower expression of miR-382-5p after the detoxification.

**Conclusions:**

Our findings identify a potential panel of peripheral markers associated with migraine subtypes and disease severity. CGRP levels as well as miRNAs expression were influenced by MO, and modulated by detoxification in subjects with CM-MO.

**Trial registration:**

The study protocol was registered at www.clinicaltrials.gov (NCT04473976).

## Background

Chronic migraine (CM) is a highly disabling condition that frequently manifests as a negative evolution of episodic migraine (EM) in a process that takes place over years [1]. In a recent meta-analysis on the predictors of migraine chronification, a number of monthly headache days ≥ 10 showed the strongest level of evidence, with depression and low household income being supported by moderate evidence [2]. In the same meta-analysis, medication overuse (MO) was associated to the highest risk ratio in a random-effects model (RR 8.82; 95% CI, 2.88–27), although the strength of evidence was rated ‘very low’due to substantial heterogeneity among studies.

It is likely that CM is the result of the dynamic interaction of multiple co-factors acting on a substrate represented by a more aggressive type of migraine. This hypothesis would explain why as many as 75% of CM subjects can spontaneously fluctuate between a chronic and an episodic pattern [3]. On the other hand, multiple pieces of evidence show that withdrawal from overused medications induces a clinically meaningful improvement in a large percentage of subjects, with rates of benefit too high to be explained by a simple placebo effect [4, 5].

The mechanisms underlying the negative effect of MO in migraine outcome are largely unknown. Multiple neural mechanisms - pronociceptive facilitation, weakened descending pain inhibition and trigeminal hyperexcitability - may be involved [1, 6]. The loss of diffuse descending inhibition has been demonstrated in a preclinical model of CM with MO (CM-MO) [7]. In this frame, it is worth noting that we have recently reported a derangement of the endocannabinoid system in subjects with CM-MO [8], which was more marked when compared with subjects suffering from EM. Interestingly in CM-MO, the clinical improvement observed after detoxification, namely MO resolution and reduction in headache frequency, is paralleled by the normalization of pain modulation mechanisms and an attenuation of central sensitization, as measured by an increase in the threshold of the nociceptive withdrawal reflex [9]. Pre-clinical studies show that triptans, the specific acute migraine drugs, induce a condition of hyperalgesia when administered chronically [10]. Thus, it is possible that acute migraine medications, when taken too frequently, may amplify the consequences of nociceptor activation and increase the probability of subsequent migraine attacks, together with the risk of MO.

In this context of multiple concurrent causes, it seems extremely important to investigate in more depth the mechanisms that may be involved in CM-MO. Calcitonin gene-related peptide (CGRP) undoubtedly plays an important, though not exclusive, role in the generation of migraine headache. CGRP receptors are localized in the anatomic sites involved in migraine pathogenesis. CGRP is involved in mast cell degranulation, neurogenic inflammation, and vasodilation [11]. It has been shown that CGRP induces IL-6 gene expression in macrophages by upregulation of circular RNA_007893, a modulator of microRNA-485-5p [12]. microRNAs (miRNAs) are involved in the generation and maintenance of chronic pain and several lines of evidence suggest that specific miRNAs may play a role in migraine pain [13–15]. In a previous clinical study, Andersen and colleagues [15] found an increased expression of miR-34a-5p during migraine attacks, while miR-382-5p levels were increased also in the attack-free phase. In addition, the peripheral expression of miR-34a-5p decreased in the saliva of young migraineurs patients under drug treatment, thus suggesting a possible role in the prediction of the therapeutic response [13]. At present, no reliable individual biomarker of migraine and its subtypes has been identified, though multiple molecules have been proposed and supported by promising results [16].

In order to provide further insights on the mediators involved in migraine pathophysiology and chronification, in this study we assayed the plasma levels of CGRP and the expression of miRNAs in peripheral blood mononuclear cells (PBMCs) of patients with EM and CM-MO. As a secondary outcome, we evaluated the changes in CGRP and miRNAs levels after detoxification in the subjects with CM-MO to gather more insights into the mechanisms that are involved in the improvement of migraine pattern following the withdrawal of the overused medications.

## Methods

### Study design

This is a cross-sectional observational controlled study with two groups (EM and CM-MO), integrated with a prospective open label interventional trial to assess the effect of detoxification in the CM-MO group on the biomarkers of interest. Samples were labeled with numerical codes and all biochemical determinations were performed by researchers who were blind to the diagnosis (RG, CD and AMZ).

### Subjects

Twenty-seven subjects with EM and 28 subjects with CM-MO were consecutively enrolled among patients attending the outpatient clinics of the Headache Science Centre of the IRCCS Mondino Foundation of Pavia (Italy).

Inclusion criteria for EM subjects were: i) diagnosis of migraine without aura according to ICHD-3 criteria [17]; ii) documented history of EM for at least 10 years before enrollment; iii) negative life-time history of CM. Inclusion criteria for CM-MO patients were: i) diagnosis of CM and MO according to ICHD-3 criteria; ii) documented pattern of stable CM in the 5 years prior to enrollment, without any remission period. This latter point was verified with a high degree of confidence by combining the information obtained from the patients’ history with their medical records, including their headache diaries.

At baseline (T0), all patients underwent a visit with a neurologist of the Headache Science Centre during which clinical/demographic data were recorded and inclusion/exclusion criteria were verified. If the criteria were met, subjects underwent peripheral venipuncture for the evaluation of CGRP, miR-382-5p, and miR-34a-5p levels.

Patients with CM-MO were hospitalized for a 7-day standardized detoxification protocol, consisting in abrupt withdrawal of overused drugs associated to intravenous therapy twice daily (08:00 a.m. and 4:00 p.m.) with isotonic 0.9% NaCl saline 500 ml + cyanocobalamin 2500 mcg + folic acid 0.70 mg + nicotinamide 12 mg + ascorbic acid 150 mg + sodic glutathione 600 mg + delorazepam 0.5 mg [4]. Two months after hospital discharge (T1), the CM-MO patients returned for a follow-up visit, during which clinical data were recorded and a second venous blood sample obtained from their ante-cubital vein.

At all study points, the patients were tested in an inter-ictal phase, defined as: 1) no headache or headache with non-migraine features and mild intensity (less than 4 on 0 to 10 visual analogue scale) in the 24 h before blood sampling; and 2) no intake of acute anti-migraine medications in the 24 h before blood sampling.

The study was approved by the local Ethics Committee (p-20170023682) and all subjects signed a written informed consent upon enrolment, in accordance with the Declaration of Helsinki and existing national ethics regulation. The study protocol was registered at www.clinicaltrials.gov (NCT04473976).

### CGRP plasma levels assay

Blood (3 ml) sampled from the cubital vein of each patient (in the interictal period, between 8:00 a.m. and 12:00 a.m.) was put into a 5 ml lavender tube (BD vacutainer TM, Becton Dickenson, Plymouth, UK). Plasma samples were prepared by centrifugation (2000 rpm for 15 min) and then stored at − 80° C. Plasma CGRP alpha levels were measured using a commercial enzyme-linked immuno-sorbent assay kit (Human α-CGRP ELISA kit, Antibodies online, Aachen, DE). Duplicate measurements were performed for each sample and the average value was calculated and considered for the analysis.

### Peripheral blood mononuclear cell (PBMC) isolation and miRNA expression

At the time of enrollment, after overnight fasting, samples of blood (18 mL) from the cubital vein were collected in sterile tubes from subjects in the interictal period, between 8:00 and 12:00 am.

miRNA expression was evaluated by real-time reverse transcription (RT) PCR in peripheral blood mononuclear cell (PBMCs). PBMCs were isolated immediately after blood collection. Briefly, blood samples were collected within ethylenediamine tetra-acetic acid (EDTA) containing tube and diluted in 1:1 ratio with phosphate buffer saline (PBS) (Sigma Aldrich, Milan, Italy). Then, diluted blood samples were slowly loaded into Ficoll separating solution (15 ml) (Sigma Aldrich, Milan, Italy) and centrifuged at 800 g for 30 min at room temperature. PBMCs accumulated as the middle white monolayer, were washed twice in sterile PBS at 300 g for 15 min. After washing, PBMCs pellet was resuspended in trizol (Bio-Rad, Milan, Italy) and stored at − 80° C until use. Total RNA (including all small non-coding RNAs) was extracted from pellets within 2 weeks using the Direct-zol RNA Mini prep plus (Zymo Research from Aurogene, Rome, Italy); then, miRNAs analysis was performed. RNA quality was determined by an optical density (OD) 260/280 ratio ≥ 1.9 and OD 260/230 ratio ≥ 1.5 by using a NanoDrop Spectrophotometer (Nanodrop™ Thermo Fisher Scientific, Euroclone Milano, Italy). Synthesis of cDNA was performed by using MirXMirna First strand Synthesis (Takara-Diatech Labline, Jesi-Ancona, Italy) and TB Green q-Rt PCR was used (Takara-Diatech, Labline Jesi-Ancona, Italy) to determine expression levels of miRNA-34a-5p and miRNA-382-5p. miRNAs expression was normalized with U6 (a type of small nuclear RNA), used as housekeeping gene. All primers of miRNAs were selected from the Prime 3 software and synthesized by Sigma Aldrich (Milan, Italy). Triplicate reactions were averaged for each miRNA. The cycle threshold (Ct) determination of the two miRNAs ranged from 25 to 28. U6 expression showed Ct values ranging from 19 to 20.

The thermocycling conditions were: 95° C for 10 min, and 40 cycles of 15 s at 95° C, followed by 1 min at 60° C by a Light Cycler 480 Instrument RT-PCR Detection System (Roche, Milan, Italy). miRNAs levels were calculated according to 2^−∆∆Ct^ = 2 − ^(∆Ct gene − ∆Ct housekeeping gene)^ formula by using Ct values.

### Statistical analysis

Based on a previous study [18], we hypothesized a meaningful difference in peripheral CGRP levels between CM-MO and EM patients of at least 50.0 ± 60.0 pg/ml (effect size of 0.981). Therefore, for a statistical power of 80%, a risk of 5% for type 1 errors, and a between groups ratio of 1:1, we calculated a sample size of at least 46 patients (23 subjects with EM and 23 subjects with CM-MO). In order to control for possible drop-outs and methodological variability when compared to previous published results [18], we aimed to enroll a population of at least 50 subjects.

The statistical analysis was performed with “R: A language and environment for statistical computing” (R Foundation for Statistical Computing, Vienna, Austria), Version 1.2.5033, for Windows. Shapiro-Wilk test showed a non-normal distribution of the biochemical variables, therefore we used non-parametric evaluations for all the analysis. Data are presented as “mean ± standard deviation” for continuous variables, and as “absolute values (percentage)” for categorical variables. Only miRNAs levels are expressed as “mean ± standard error of the mean”, to allow comparison with previous published data [15]. For continuous variables, differences between groups were analyzed using the Mann-Whitney U test, while for categorical variables, statistical analysis was performed with the chi-square test. Correlation analysis was performed by means of the Spearman test. Finally, we performed a logistic regression with the EM and CM-MO as dependent variables, using as covariates the three biochemical dosages as well as other clinical and demographic variables of interest according to the univariate analysis. For all the previously described analyses, the levels of significance were corrected with a Bonferroni method to account for multiple comparison.

To evaluate the role of detoxification in CM-MO patients, we used non-parametric tests for repeated measures [19] with two factors: factor TIME (T0 vs. T1), and factor GROUP (responders vs. non-responders). Patients were qualified as “responders” if they achieved at T1 a reduction in monthly migraine days of at least 30% during the previous month, according to the official guidelines for clinical trials in CM of the International Headache Society [20]. Differences between groups were analyzed using the Mann-Whitney U test or the Kruskal-Wallis test. The levels of significance were further corrected with a Bonferroni method to account for multiple comparisons. For all the performed tests, the level of significance was set at α = 0.050.

## Results

### Baseline clinical and demographic features of study population

EM patients (*n =* 27; 39.2 ± 8.8 years; 92.6% females) reported 6.9 ± 2.4 monthly migraine days, and they were taking acute medications on 6.7 ± 2.2 days per month. CM-MO patients (*n = 28*; 47.6 ± 10.9 years; 85.7% females) reported 23.6 ± 5.3 monthly migraine days, and they were taking acute medications on 23.3 ± 5.6 days per month. A percentage of 32.1% of patients had already undergone at least one detoxification in the past.

CM-MO patients were significantly older than EM patients (*p* = 0.002), and their migraine duration (30.8 ± 12.1 years) was significantly longer when compared to EM patients (22.6 ± 9.7 years) (*p* = 0.013). Clinical and demographic data are summarized in Table 1.

**Table 1:** Clinical and demographic features of study population at baseline

	EM	CM-MO	***p***-value
***n***	27	28	–
**Age**	39.2 ± 8.8	47.6 ± 10.9	0.002
**Sex (female)**	25 (92.6%)	24 (85.7%)	0.669
**Duration of migraine (years)**	22.6 ± 9.7	30.8 ± 12.1	0.013
**Duration of chronic migraine (years)**	–	12.2 ± 10.8	–
**Duration of medication overuse (years)**	–	8.6 ± 6.4	–
**Migraine days per month**	6.9 ± 2.4	23.6 ± 5.3	0.001
**Headache days per month**	7.2 ± 2.4	26.2 ± 5.2	0.001
**Days of acute drug intake per month**	6.7 ± 2.2	23.2 ± 5.6	0.001
**Doses of acute drugs per month**	7.2 ± 2.7	51.5 ± 60.5	0.001
**Migraine with aura**	5 (18.5%)	7 (25.0%)	0.746
**Preventive therapy at baseline**	10 (37.0%)	10 (35.7%)	1.000
**Previous detoxification**	–	32.1%	–
**Acute treatment**			
**NSAID**	13 (48.1%)	14 (50.0%)	0.845
**Triptan**	8 (29.6%)	7 (25.0%)	
**Combination**	4 (14.8%)	6 (21.4%)	
**Politherapy**	2 (7.4%)	1 (3.6%)	

*Legend*: *EM* episodic migraine, *CM-MO* chronic migraine with medication overuse, *NSAID* nonsteroidal anti-inflammatory drugs. Continuous variables are presented as mean ± standard deviation

### CGRP and miRNAs profiles in EM and CM-MO

**Fig. 1:**
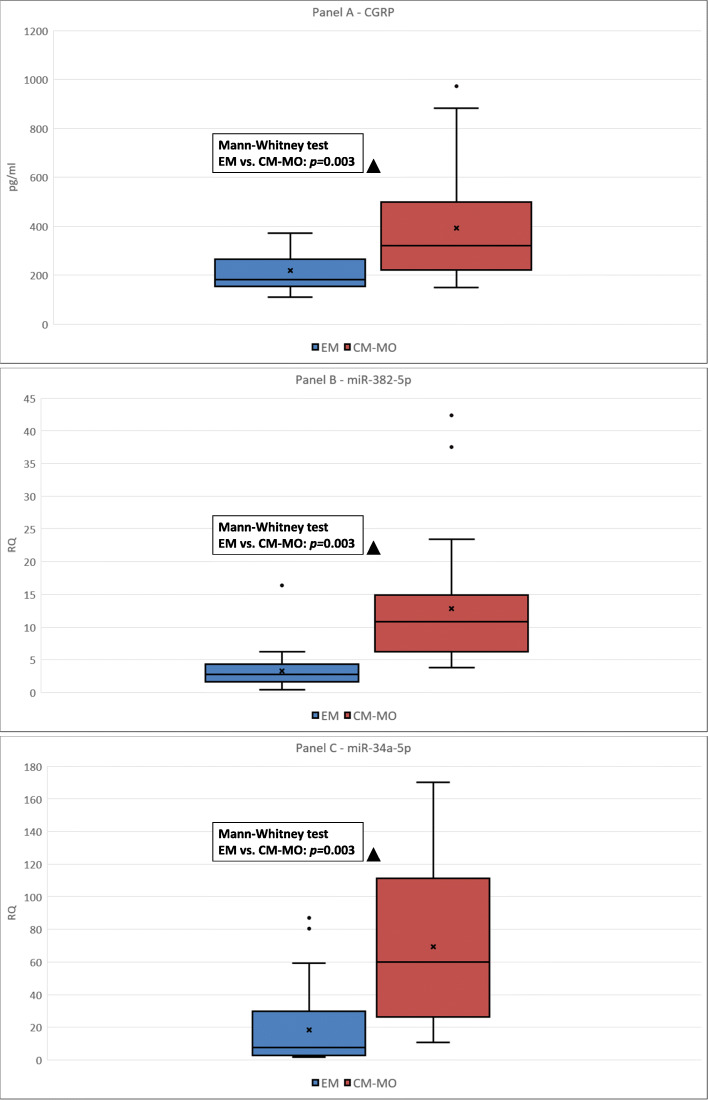
CGRP plasma levels, and miR-382-5p and miR-34a-5p expression in peripheral blood mononuclear cells of subjects with episodic migraine and chronic migraine with medication overuse. EM: episodic migraine (*n* = 27); CM-MO: chronic migraine with medication overuse (*n* = 28*).* Panel **a**: comparison of CGRP between EM and CM-MO subjects. Panel **b**: comparison of miR-382-5p between EM and CM-MO subjects. Panel **c**: comparison of miR-34a-5p between EM and CM-MO subjects. Box-plots: the distance between the top and bottom of the box represents the interquartile range (IQR: 25th percentile to 75th percentile). The line inside the box represents the median value, while the cross inside the box represents the mean value. The upper and lower whiskers represent the maximum and minimum values respectively (outliers excluded). Black dots above the upper whiskers represent possible outliers (outliers are statistically defined as values outside the 75th percentile + 1.5*IQR). Δ: EM vs. CM-MOH: *p* < 0.005 (Mann-Whitney Test with post-hoc Bonferroni’s correction). RQ: Relative quantification: 2^−∆∆Ct^ = 2 − ^(∆Ct gene − ∆Ct housekeeping gene)^; Ct: cycle threshold

CGRP plasma levels were higher in the CM-MO group (393.3 ± 242.9 pg/mL) when compared to EM patients (220.4 ± 83.42 pg/mL) (*p* = 0.003). The relative expression (RQ) of miR-382-5p in PBMCs was higher in the CM-MO group (14.6 ± 3.2) when compared to EM patients (3.3 ± 0.6) (*p* = 0.003). This was also the case for miR-34a-5p expression, which was higher in CM-MO patients (69.5 ± 8.5) than in EM subjects (18.4 ± 4.7) (*p* = 0.003). CGRP plasma levels and miRNAs expression in PBMCs of EM and CM-MO groups are illustrated in Fig. 1.

CGRP plasma levels and relative expression of miR-382-5p and miR-34a-5p in PBMCs were not associated to gender, use of preventive therapy or concomitant diagnosis of migraine with aura.

### Correlation analysis

In the overall population, CGRP plasma levels positively correlated with miR-382-5p (Spearman’s rho: 0.491, *p* = 0.001) and miR-34a-5p (Spearman’s rho: 0.303, *p* = 0.025) expression in the PBMCs. Moreover, CGRP, miR-382-5p, and miR-34a-5p levels correlated positively with monthly migraine days, monthly headache days, days of intake of acute drugs and monthly doses of acute drugs (*p* < 0.05 for all comparison). miR-34a-5p levels were also correlated with age (Spearman’s rho: 0.321, *p* = 0.017).

After correction for age, sex, and disease duration using a logistic regression analysis, we found that miRNAs levels were still significantly associated with the migraine phenotype EM/CM-MO (*p* = 0.014 for miR-382-5p, and *p* = 0.038 for miR-34a-5p), while CGRP levels were not (*p* = 0.115). The highest effect was recorded for miR-382-5p, with an odds ratio of 1.794 (95% confidence interval of 1.124–2.862) for unitary increase in favour of the diagnosis of CM-MO (Table 2). Although a significant correlation was present between CGRP and miRNAs levels, we did not find significant collinearity in the linear regression model.

**Table 2:** Results of the logistic regression analysis for dependent variables: EM vs. CM-MO

	B	S.E.	Wald	df	*p*-value	Exp(B)	95% C.I. for Exp(B)	
Age	− 0.164	2.116	1.983	1	0.159	0.849	0.676	1.066
Sex (male)	−0.150	1.538	0.010	1	0.922	0.861	0.042	17.532
Disease duration	0.221	0.131	2.845	1	0.092	1.247	0.965	1.612
CGRP	0.008	0.005	2.485	1	0.115	1.008	0.998	1.017
miR-382-5p	0.584	0.238	6.005	1	**0.014**	1.794	1.124	2.862
miR-34a-5p	0.032	0.015	4.322	1	**0.038**	1.033	1.002	1.064
Constant	−6.150	2.487	6.118	1	0.013	0.002	–	–

*Legend*: *EM* episodic migraine, *CM-MO* chronic migraine with medication overuse. B: coefficients (log-odds units) for the logistic regression equation for predicting the dependent variable (EM vs. CM-MO) from the independent variable. *S.E.* standard errors associated with the coefficients, *Wald* Wald chi-square value, *df* degrees of freedom. Exp(B): odds ratios for the predictors (exponentiation of the coefficients). *95% C.I. for Exp(B)* 95% confidence intervals for Exp(B)

When the correlation analysis was repeated separately for the EM and CM-MO subgroups, we did not find any significant correlations in the CM-MO group, while in the EM group we obtained a positive and significant correlation between CGRP and miR-382-5p (Spearman’s rho: 0.527, *p* = 0.005).

### Effects of detoxification from overused drugs in CM-MO patients

#### Clinical outcomes

All the CM-MO subjects completed the one-week detoxification protocol and returned for the follow-up visit after 2 months (T1). When compared to baseline, at T1 we observed a significant reduction in monthly migraine days (23.6 ± 5.3 vs. 9.6 ± 7.3; *p* = 0.001), monthly headache days (26.9 ± 5.2 vs. 13.4 ± 9.9; *p* = 0.001), monthly days of intake of acute medications (23.3 ± 5.6 vs. 8.8 ± 7.7; *p* = 0.001) and monthly doses of acute medications (51.5 ± 60.5 vs. 11.2 ± 11.3; *p* = 0.001).

Eighty-two % of patients (*n = 23*) qualified as “responders” after 2 months from detoxification, as previously defined, while the remaining 5 patients were “non-responders”. Responder and non-responder groups were comparable for clinical and demographic features at baseline. In particular, these groups did not differ at baseline in terms of migraine days (responders: 23.8 ± 5.2; non-responders: 22.8 ± 6.7; *p* = 0.816), headache days (responders: 26.0 ± 5.1; non-responders: 27.2 ± 6.3; *p* = 0.482), days of intake of acute medications (responders: 23.5 ± 5.4; non-responders: 22.2 ± 7.4; *p* = 0.816) and doses of acute medications (responders: 40.5 ± 31.3; non-responders: 102.2 ± 124.0; 0.727). At T1, responders reported 7.0 ± 4.4 migraine days/month, 11.2 ± 9.5 headache days/month, 5.9 ± 4.5 days of intake of acute medications/month, and 7.0 ± 5.5 doses of acute medications/month. Non-responders reported 21.8 ± 5.1 migraine days/month, 23.4 ± 4.0 headache days/month, 22.0 ± 4.9 days of intake of acute medications/month, and 30.4 ± 11.7 doses of acute medications/month. At T1, all the above parameters were significantly different between responders and non-responders, *p* < 0.020).

#### CGRP and miRNAs profiles

At baseline, there were no significant differences between responders and non-responders as regards miR-382-5p and miR-34a-5p expression (*p* = 0.186, and *p* = 1.000, respectively). In contrast, CGRP levels were higher in non-responders when compared to responders (*p* = 0.036).

**Fig. 2:**
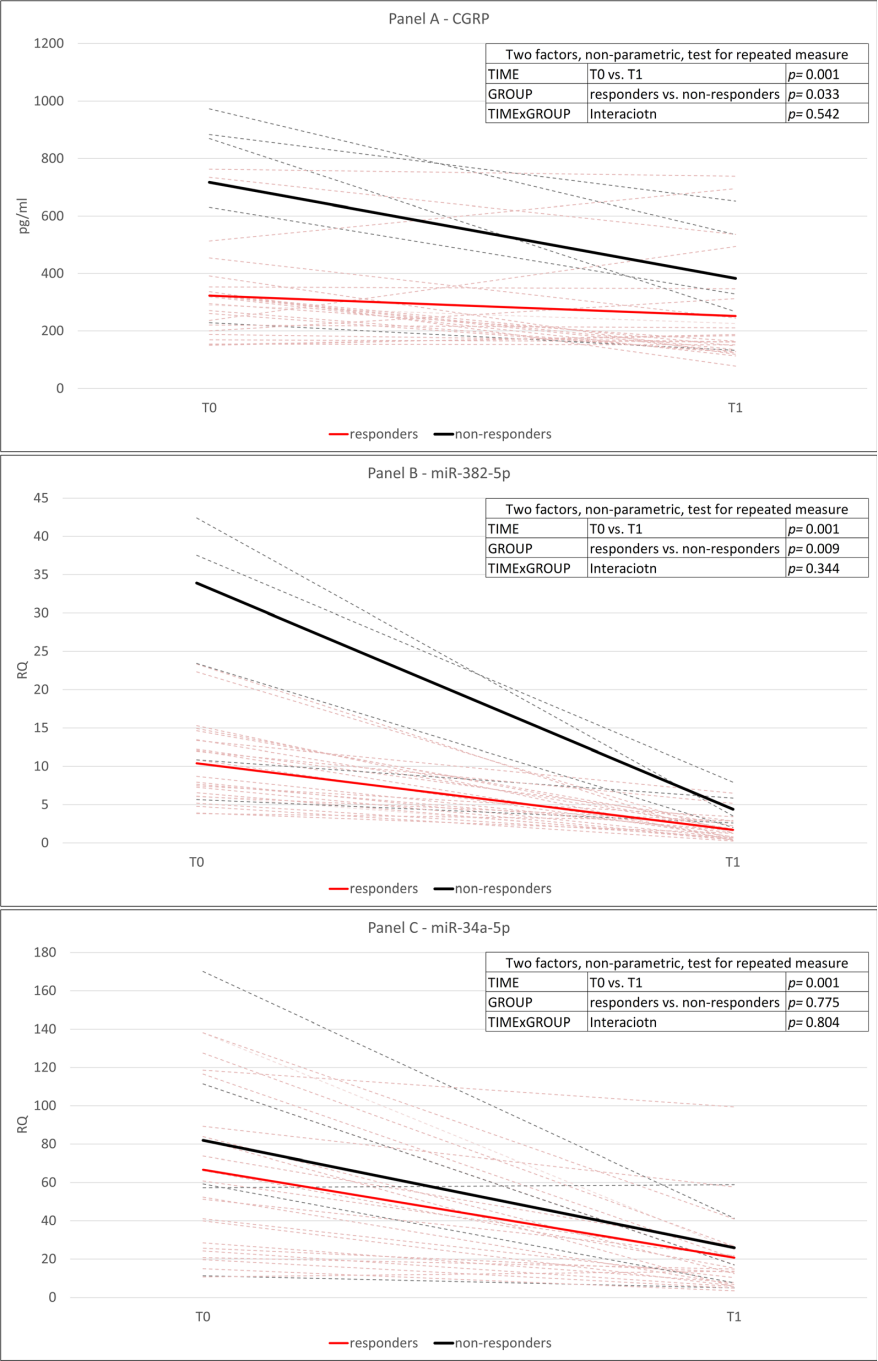
Changes in CGRP plasma levels and miR-382-5p and miR-34a-5p expression in peripheral blood mononuclear cells of subjects with chronic migraine with medication overuse treated with a detoxification protocol. Study population: *n* = 28; 30% responders: *n* = 23; 30% non-responders: *n* = 5. PBMCs: peripheral blood mononuclear cells. Panel **a**: changes in CGRP plasma levels after detoxification. Panel **b**: changes in miR-382-5p expression in PBMCs after detoxification. Panel **c**: changes in miR-34-5p expression in PBMCs after detoxification. The box in the panels highlights the results of statistical analysis performed with two factors, non-parametric test for repeated measures: factor TIME: T0 vs. T1; factor GROUP: 30% responders vs. non-responders. Dashed lines represent estimates for each patient (red: 30% responders; black: 30% non-responders). Continuous lines represent the mean of the group (red: 30% responders; black: 30% non-responders). T0: baseline evaluation; T1: 2 months after discharge from in-hospital detoxification. RQ: Relative quantification*:* 2^−∆∆Ct^ = 2 − ^(∆Ct gene − ∆Ct housekeeping gene)^; Ct: cycle threshold as defined in methods section

After detoxification (T1), CGRP levels as well as expression of miR-382-5p, and miR-34a-5p decreased significantly in the overall population (factor TIME “T0 vs. T1”: *p* = 0.001 for all the three biochemical variables). After detoxification, the pattern of modifications of the three biochemical variables did not differ between responders and non-responders, as suggested by the no significant interaction TIMExGROUP for CGRP (*p* = 0.542), miR-382-5p (*p* = 0.344) and miR-34a-5p (*p* = 0.804). CGRP and miRNAs data for CM-MO subjects before and after detoxification are depicted in Fig. 2, and summarized in Table 3.

**Table 3:** Biochemical data of CM-MO patients before and after detoxification

	T0			T1			Statistical analysis		
	All patients	Responders	Non-responders	All patients	Responders	Non-responders	TIME	GROUP	TIME x GROUP
*n*	28	23	5	28	23	5	–		
CGRP (pg/ml)	393.3 ± 242.9	322.9 ± 164.1	717.1 ± 301.1	275.2 ± 251.8	251.8 ± 383.2	383.2 ± 208.7	0.001	0.033	0.542
miR-382-5p (RQ)	14.6 ± 3.2	10.4 ± 1.1	33.9 ± 15.6	2.2 ± 0.4	1.7 ± 0.3	4.4 ± 1.1	0.001	0.009	0.344
miR-34a-5p (RQ)	69.5 ± 8.5	66.7 ± 8.8	81.8 ± 27.2	21.6 ± 4.0	20.7 ± 4.4	25.9 ± 10.4	0.001	0.775	0.804

*Legend*: *CM-MO* chronic migraine with medication overuse. Responders: patients with a reduction of at least 30% of monthly migraine days after detoxification. T0: baseline evaluation; T1: 2 months after discharge from in-hospital detoxification. CGRP values are presented as mean ± standard deviation. microRNAs values are presented as “mean ± standard error of the mean”. *RQ* Relative quantification: 2^−∆∆Ct^ = 2 − ^(∆Ct gene − ∆Ct housekeeping gene)^, *Ct* cycle threshold as defined in methods section. Statistical analysis: non-parametric tests for repeated measures with two factors: factor TIME (T0 vs. T1), and factor GROUP (responders vs. non-responders)

It is worth noting that, at T1, the levels of CGRP and the expression of miR-34a-5p observed in responders and non-responders were comparable to those of EM patients (*p* = 0.472, and *p* = 0.248, respectively), while the expression of miR-382-5p was significantly lower (*p* = 0.006). This latter difference was mainly explained by the pronounced reduction of miR-382-5p expression recorded in responders (responders vs. EM: *p* = 0.042; responders vs. non-responders: *p* = 0.034; non-responders vs. EM: *p* = 1.000) (Table 4).

**Table 4:** Comparison of biochemical variables between EM and CM-MO patients after detoxification

	EM	CM-MO at T1			Kruskal-Wallis test	Post-hoc		
		All patients	Responders	Non-responders		Responders vs. EM	Responders vs. non-responders	Non-responders vs. EM
*n*	27	28	23	5		–		
CGRP (pg/ml)	220.4 ± 84.4	275.2 ± 251.8	251.8 ± 383.2	383.2 ± 208.7	0.472	–	–	–
miR-382-5p (RQ)	3.3 ± 0.63	2.2 ± 0.4	1.7 ± 0.3	4.4 ± 1.1	0.006	0.042	0.034	1.000
miR-34a-5p (RQ)	18.4 ± 4.7	21.6 ± 4.0	20.7 ± 4.4	25.9 ± 10.4	0.248	–	–	–

*Legend*: *EM* episodic migraine, *CM-MO* chronic migraine with medication overuse. Responders: patients with a reduction of at least 30% in monthly migraine days after detoxification. CGRP values are presented as mean ± standard deviation. microRNAs values are presented as mean ± standard error of the mean. *RQ* Relative quantification*:* 2^−∆∆Ct^ = 2 − ^(∆Ct gene − ∆Ct housekeeping gene)^, *Ct* cycle threshold as defined in methods section. Statistical analysis: differences among EM groups, responders, and non-responders patients were analyzed with Kruskal-Wallis test followed by a post-hoc analysis; the levels of significance were further corrected with a Bonferroni method to account for multiple comparison

## Discussion

Chronification represents a critical event in the evolution of migraine, as it leads to increased disability and loss in quality of life. Migraine chronification therefore deserves specific scientific attention aimed at identifying the mechanisms and mediators involved.

CGRP is involved in trigeminovascular activation and it is likely to play a role in migraine chronification [21]. CGRP contributes to the release of pro-nociceptive substances from trigeminovascular terminals and to the enhancement of neuronal activity leading to central sensitization [22, 23]. Peripheral levels of CGRP have been proposed as a potential marker of CM, based on the higher values observed interictally, in the absence of symptomatic medications [24]. Plasma CGRP levels may represent a reliable marker of CGRP activity at the receptor level. This hypothesis could explain the high tolerability of the novel anti-CGRP monoclonal antibodies in migraine sufferers. Indeed, CGRP is still present in the plasma of patients chronically treated with erenumab, supporting the idea that a physiological receptor activity is still preserved despite a significant preventive efficacy [25].

Several data on miRNAs expression have shown an association with EM and chronic pain [15, 26]. However, to the best of our knowledge, no study has investigated the role of miR-382-5p and miR-34-5p in CM.

The main finding of this study is that the levels of CGRP and miRNAs were significantly higher in CM-MO subjects when compared to EM patients. CGRP and miRNAs levels were also positively correlated with monthly migraine days, monthly headache days, days of intake of acute drugs and doses of acute drugs. It is worth noting that our study population comprises patients with a conspicuous assumption of acute anti-migraine drugs, namely with a ratio between migraine days and days of acute drug intake around 1. This is relevant because the almost perfect parallelism between migraine days and days of acute drug intake in our population of CM-MO subjects prevents the possibility to disentangle the role of the respective variables in the observed results. In this frame, it is worth noting that miR-34a-5p expression decreased in the saliva of migraineurs under drug treatment (acute NSAIDs + chronic magnesium), compared with untreated migraineurs [13]. Alterations in miRNAs levels may also reflect reversible alterations in gene expression that occur in response to environmental factors, such as inflammation. miRNAs may play a function in pathological processes, and changes in their regulation are associated with several neurological diseases, including drug addiction [27].

On the other hand, it is important to observe that, after a multivariate analysis with correction for age, sex, and disease duration, miRNAs levels were still significantly associated with migraine phenotype (EM vs. CM-MO) (*p* = 0.014 for miR-382-5p, and *p* = 0.038 for miR-34a-5p), while CGRP plasma levels were not (*p* = 0.115). This latter finding, together with the observed reduction in CGRP after the detoxification, suggests that CGRP levels in migraineurs may reflect multiple factors: duration of the disease, clinical phenotype (episodic/chronic) and intake of acute medications. The conflicting data from the literature on peripheral CGRP levels in EM and CM does not offer solid clues for further elaboration on this finding. Lee et al. found that the interictal serum levels of CGRP were not elevated in CM subjects, and that the use of preventive medications did not influence CGRP levels [28]. The Danish Headache Group [29] reported no difference in CGRP serum levels in CM-MO patients compared with healthy volunteers either before or after detoxification. By contrast, Cernuda-Morollón and co-workers [18] found increased CGRP serum levels in CM women with and without analgesic overuse, compared to EM and healthy controls, thus suggesting a possible pathophysiological mechanism for CGRP in migraine chronification. In line with this hypothesis, CGRP plasma levels were also found significantly elevated in migraineurs requiring preventive therapy [30], suggesting that patients with higher CGRP plasma levels may suffer more frequent migraine attacks. Technical and methodological factors may have contributed to the heterogenicity of results, such as differences in sex distribution, age, specimen source (serum vs plasma), participant selection, timing of blood sample collection and assay methods used (ELISA vs RIA, CGRP alpha isoform vs CGRP beta isoform) [31].

Interestingly, we showed that CGRP plasma levels positively correlated with the peripheral level of both miRNAs. This observation suggests an interaction between CGRP and these signaling molecules. The biological pathways through which the interaction may take place are elusive at this time, but it is possible to hypothesize an inflammation-related mechanism, as suggested by the observation that CGRP induces IL-6 mRNA expression in macrophages by an indirect modulation of miR-485-5p [12].

A further piece of information was provided by the results obtained after detoxification in the CM-MO subjects: the treatment induced a decrease in CGRP, miR-382-5p, and miR-34a-5p levels. This finding strongly suggests a role for medication overuse in the biomolecular pathways under investigation. Our findings did not show a clear separation of the effect of detoxification in responder and non-responder groups. Caution is needed in the interpretation of this negative finding because the number of non-responders to detoxification was very small (*n* = 5). However, our findings support the notion that our one-week detoxification protocol and the subsequent reduction of doses of acute medications (a pattern observed also in the non-responder group, though not to a statistically significant level) had an impact on peripheral levels of CGRP and miRNAs, even if it did not translate into a clinically relevant improvement in all the subjects. In this frame, it is interesting to note that a similar pattern of reduction in the levels of the same miRNAs was detected in CM subjects treated with erenumab regardless of their clinical improvement [32], which suggests that changes in biomolecular pathways implicated in migraine may take place in the absence of a clinical meaningful response. Thus, we hypothesize that, although the levels of miRNAs seem to be strictly related to migraine and its severity, they are not sensitive enough to act as predictors of the clinical improvement [32]. This said, the non-responders to the detoxification are defined by a specific biochemical profile at baseline, characterized by significantly higher levels of CGRP and a tendency toward higher expression of miR-382-5p. This result must be interpreted cautiously due to several reasons: our study was not powered to test this specific hypothesis; the number of non-responders is low (*n* = 5), which does not allow further statistical evaluations, such as intra-group correlation analysis; previous data from literature does not support our finding. Indeed, Munksgaard et al. [29] did not detect significant changes in the peripheral levels of CGRP after detoxification of subjects with MO headache, despite a marked reduction in headache frequency. It must be noted that in their study, the group of subjects included in the post-detoxification evaluation was quite small (*n* = 10) and MO headache was not limited to subjects with migraine as primary headache.

Finally, it is worth noting that the detoxification program reduced CGRP, miR-34a-5p, and miR-382-5p in the CM-MO patients to levels comparable to the EM group, which further underscores the possibility that acute medication overuse actually modifies the biology of migraine.

Some limitations must be acknowledged in the interpretation of our results. Firstly, our study did not include a group of healthy controls. This choice was made to improve the study feasibility, but also because, due to the lack of preliminary data, we first needed to probe the actual existence of an interaction between CGRP and investigated miRNAs. Another limitation is that our study was not primarily powered to test the effects of detoxification and so, although interesting, these results need confirmation in specifically targeted studies. The detoxification protocol indeed proved effective in the large majority of our CM-MO patients, with only 5 out of 28 subjects being non-responders, which ultimately led to numerically imbalanced subgroups and thus prevents generalization of our results.

## Conclusions

The present findings point to a panel of biomolecular markers of migraine subtypes represented by CGRP and two miRNAs. CGRP and miRNAs are associated with each other at the individual level across the migraine spectrum. The peripheral levels of the molecules correlated with the clinical indicators of migraine severity, being higher in CM-MO as compared to EM. Detoxification from medication overuse decreased the peripheral levels of these molecules in the group of subjects with CM-MO. The reported changes provide interesting evidences for a biological effect of detoxification and pave the way for future, larger and specifically targeted, investigations for the characterization and validation of migraine biomarkers, a necessary step for developing personalized therapeutic approaches.

## Data Availability

The datasets used and/or analysed during the current study are available from the corresponding author on reasonable request.

## Abbreviations

EM: Episodic migraine
CM-MO: Chronic migraine with medication overuse
CGRP: Calcitonin gene-related peptide
PBMC: Peripheral blood mononuclear cell
